# Amplifications of stemness genes and the capacity of breast tumors for metastasis

**DOI:** 10.18632/oncotarget.27608

**Published:** 2020-05-26

**Authors:** Nikolai Litviakov, Marina Ibragimova, Matvey Tsyganov, Polina Kazantseva, Irina Deryusheva, Alina Pevzner, Artem Doroshenko, Eugeny Garbukov, Natalia Tarabanovskaya, Elena Slonimskaya

**Affiliations:** ^1^Laboratory of Oncovirology, Cancer Research Institute Tomsk NRMC, Tomsk, Russia; ^2^Biological Institute of National Research Tomsk State University, Tomsk, Russia; ^3^Department of General Oncology, Cancer Research Institute Tomsk NRMC, Tomsk, Russia; ^4^Faculty of Medicine, Department of Oncology, Saint-Petersburg State University, Saint-Petersburg, Russia

**Keywords:** stemness genes, cancer stem cells, breast cancer, microarray analysis, metastasis-free survival

## Abstract

Introduction: The phenomenon of non-CSC (cancer stem cell) to CSC plasticity has been previously described in multiple studies and occurs during the ectopic expression of stemness genes such as *OCT3*, *SOX2*, *KLF4*, *MYC*, *NOTCH1*, and *NANOG*. In our opinion, acquiring the ability to ectopically express stemness genes, selected by bioinformatics analysis and, accordingly, non-CSC to CSC plasticity, is due to amplification of genes at the following locations: 3q, 5p, 6p, 7q, 8q, 13q, 9p, 9q, 10p, 10q21.1, 16p, 18chr, 19p. This paper demonstrates the significance of stemness gene amplifications leading to metastasis and stem-like cancer cell activity.

Materials and Methods: In our studies, stemness gene amplifications were determined using the CytoScan HD Array. We studied the association of changes in stemness gene amplifications in tumors with metastasis treated with neoadjuvant chemotherapy (NAC) in 50 patients with breast cancer. We used qPCR to evaluate the expression of 13 stemness genes in tumors before and after NAC in 98 patients with breast cancer. Using primary cultures from the breast tumor of patient St23784/17 with stemness gene amplifications (*SOX2*, *MYC*, *KLF4*, *NOTCH1*, *NODAL*) and patient Ti41749/17 without stemness gene amplifications in the tumor, we studied the expression of stemness genes, proliferative tumor stem-cell activity, mammosphere formation, and expression of the CD44 tumor stem cell marker.

Results: The occurrence of amplifications at regions of stemness gene localization during NAC (22% cases) in residual tumors was associated with a very high metastasis rate (91% cases). Eliminating tumor clones with stemness gene amplifications using NAC (42% cases) led to 100% metastasis-free survival.

In patients who developed hematogenic metastases after treatment, the expression of 7/13 stemness genes in the residual tumor after NAC was statistically higher than in patients without metastases. Primary cultures of EpCam^+^ tumor cells from patients with stemness gene amplifications revealed high proliferative activity. After the 3rd passage, the number of tumor cells increased 30-fold. Due to IL-6, this cell population showed a 2.5-fold increase in the EpCam^+^CD44^hi^CD24^–/low^ and 2-fold decrease in the EpCam^+^CD44^low^CD24^–^ subpopulations of tumor stem cells; the formation of mammospheres was also observed. Primary cultures of EpCam+ tumor cells from the patient with no stemness gene amplifications had relatively low proliferative activity. IL-6 caused a 2.3-fold increase in the EpCam^+^CD44^low^CD24^–^ and 2-fold decrease in the EpCam^+^CD44^hi^CD24^–/low^ subpopulations of tumor stem cells with no induction of mammospheres.

Conclusions: The results of this study show that stemness gene amplifications in tumor cells are associated with metastasis and determine their potential stem property activation and non-CSC to CSC plasticity with the formation of EpCam^+^CD44^hi^CD24^–/low^ cells, active proliferation, mammosphere formation, and metastasis.

## INTRODUCTION

According to the hierarchical model of tumor growth, only cancer stem cells (CSCs), but not differentiated tumor cells, are capable of forming new tumors and metastases [1]. Conversely, the plasticity of differentiated tumor cells has been shown, as well as the acquisition of a stem phenotype by differentiated tumor cells [2] and a model of neutral competition [3]. For clarity, we hereinafter refer to the plasticity of differentiated tumor cells and their dedifferentiation from non-CSCs to CSCs as non-CSC plasticity. In this paper, we consider the problem of tumor cells acquiring the ability to metastasize.

In 2013, Cristine Chaffer et al. expressed the opinion that different tumors differ significantly in their non-CSC plasticity, which will determine their malignant potential and ability to progress [4]. Like Cristine Chaffer, we suspect that non-CSC plasticity, stemness induction, and stem-like cell activity determine tumor malignancy and, most importantly, the ability to metastasize. Lack of non-CSC plasticity and induction of a complete stem phenotype, as well as low stem-like cancer cell ability, explain the inability of a tumor to metastasize.

Apart from studies showing some cytokines and microRNAs that induce a stem phenotype [5], a wide range of studies have shown that forced overexpression (e. g., transfection) of stemness genes, such as *OCT3*, *SOX2*, *KLF4*, *MYC*, *NOTCH1*, *NANOG*, and *LIN28*, as well as epithelial-mesenchymal transition (EMT) genes (*ZEB1*, *SNAI*, *VIM*, *TWIST*, etc.), leads to stem phenotype induction and increased stem-like cancer cell activity [4, 6–14].

Thus, ectopic expression of stemness genes determines the possibility of non-CSC plasticity, induction of complete stem properties and activity of stem-like cancer cells (active proliferation in symmetric division and tumor-initiating properties). We suspect that the key point in the metastasis process is the acquisition of the ability to ectopically express stemness genes, which in many cases is the result of stemness gene loci amplification. On the one hand, in cases of amplification, the level of expression can increase; on the other hand, the response to stimulation of expression by external and internal factors also increases (microRNA, cytokines, other factors in the microenvironment). In addition, the mechanisms responsible for their down-regulation may be ineffective when the stemness gene loci are amplified.

The selection of stemness genes was based on bioinformatics analysis of databases, the literature and the CNA (copy number aberration)-genetic landscape of a breast tumor. The databases were used for bioinformatics analysis (https://reactome.org/PathwayBrowser/#/R-HSA-452723&PATH=R-HSA-1266738&DTAB=MT; https://www.rndsystems.com/research-area/cancer-stem-cell-transcription-factors [15]). Common genes for the EMT process and induction of pluripotent cells were also selected (http://dbemt.bioinfo-minzhao.org/dbemt2_pmid.txt). Within the first phase, 156 genes were selected. In the second phase, candidate genes were selected that were involved in the induction and maintenance of cell pluripotency based on the published literature. The selection process was conducted, and if several articles supported the role of the gene in the induction and maintenance of pluripotency, the gene was selected. Subsequently, 53 genes were selected. At the third stage, only those genes that were selected were localized in chromosomal regions with amplification in breast tumors [16]. For example, despite the well-known role of the induction of pluripotent cells, LIN28A located on the short arm of chromosome 1 in cytobend 1p36.11, in which amplification is practically absent, was excluded [16].

The Supplementary Table 1 is annotated to select stemness genes that, according to the literature, participate in the induction and maintenance of the stem phenotype and their chromosomal localization. According to our annotation, the chromosome arms where the stemness genes are localized are as follows: 3q(26.33; 34), 5p(15.33; 13.1), 6p(24.3; 22.3; 21.33; 21.32), 7q(11.23; 21.13; 31.2; 32.1), 8q(11.21; 24), 9p(21.2), 9q(34.3; 21.13; 31.2, 22.33), 10p(15.2; 13; 12.2; 11.22), 10q22.1, 12p(13.31) 13q(34; 32.3; 22.1; 13.3; 12.2), 16p(11.2; 13.3), 18q(21.1; 21.2), 19p(13.3; 13.2; 13.12). Many such amplifications occur in the tumor and are associated with adverse outcomes. According to the Progenetix database (http://www.progenetix.org/) the 8q24 amplification was the most common CNA in terms of incidence in all the studied tumor localizations (177 tumor types) and was found in more than 30% of all samples [17]. The most significant gene in this locus, according to COSMIC, is the protooncogene *MYC* [18], and it is also a stemness gene that is included in Shinya Yamanaka’s cocktail [19]. *MYC* promotes the activation of de novo enhancers, which support the activation of oncogenic pathways (e. g., *WNT* and *EGFR*) and the onset of a stem cell-like state represented by enhanced mammosphere formation [20]. The 8q region amplifications predict the development of metastases in patients with breast cancer [21]. Wikman H et al. (2012) showed a high frequency (more than 30%) of amplification at the 8q and 16p loci in the primary breast tumors of patients with brain metastases. In metastases, gains were found in many loci, including several from our list, 5p, 7, 8q, 10p, and 18, with an incidence of more than 30% of cases [22].

To address the problem of determining a tumor’s ability to metastasize, we studied the importance of the amplification of stemness genes localized on 3q, 5p, 6p, 7q, 8q, 13q, 9p, 9q, 10p,10q21.1, 16p, 18chr, and 19p for breast cancer metastasis, stem-like activity and dedifferentiation from non-CSCs to CSCs of breast tumor cells. We assume that any two or more focal amplifications of the aforementioned regions are necessary to acquire the ability for metastasis.

## RESULTS

### Changes in stemness gene amplification in breast tumors during NAC

In each of the 50 patients, we compared the CNA (CNA–copy number aberration)-genetic landscape of breast tumors before and after neoadjuvant chemotherapy (NAC), and we evaluated changes in stemness gene amplification during the NAC.

Supplementary Figure 1 presents a general overview of the CNA-landscape of all patients. Changes in stemness gene amplification during NAC have been associated with hematogenous metastasis, demonstrating their significance in the development of metastases.

Table 1 presents the generalized data for changes in amplification at regions of stemness gene localization in the process of NAC, clinical indicators of tumors of all 50 patients, NAC effects, and metastasis. Eight patients showed no amplification of stemness genes before treatment and after NAC. Six patients (G373/2, B156/5, E106/2, L188/4, S300/3, Sh244/4) had one amplification of the stemness gene localization region in their tumors before treatment. In four of six patients (B156/5, E106/2, L188/4, S300/3), NAC stimulated the formation of new tumor clones with amplifications at regions of stemness gene localization, and all patients developed metastases.

**Table 1:** Clinical and morphological parameters of patients and alteration of stemness gene amplification during NAC

Patients^#^	Amplification of stemness genes		Change during NAC	T	N	Molecularsubtype	Efficiency NAC	Metastasis	MFS, month
	Pretreatment	Postreatment							
A126/3	8q16p	no	elimination	2	1	LumB	pCR	no	24
B386/2	3q10p13q	no	elimination	2	0	LumB	pCR	no	35
G265/5	7q9p10p19p	no	elimination	3	0	LumB	pCR	no	31
I133/4	7q8q	no	elimination	2	0	LumB	pCR	no	22
Ya134/1	8q10p	no	elimination	2	0	LumB	pCR	no	32
K467/2	8q10p16p	no	elimination	2	1	LumB	pCR	no	24
Sh244/4	16p	no	elimination	2	1	LumB	PR	no	18
B394/2	8q10p	no	elimination	2	0	LumB	PR	no	35
M483/1	8q16p	no	elimination	2	1	LumB	PR	no	52
V396/2	7q16p	16p	elimination	2	1	LumB	PR	no	113
S577/2	3q6p	3q	elimination	2	0	LumB	PR	no	37
S702/2	5p8q	8q	elimination	2	1	LumB	PR	no	96
K345/2	5p8q16p	no	elimination	1	1	LumB	PR	no	97
M366/2	3q6p8q	3q8q	elimination	2	0	LumB	PR	no	48
L355/3	8q13q16p18	3q8q16p	elimination	2	0	LumB	PR	no	30
Sh198/2	7q10p10q16p	7q	elimination	2	0	TNBC	PR	no	89
L234/2	8q9p9q10p16p	8q	elimination	2	0	LumB	PR	no	56
S454/3	5p6p8q9q10p16p	no	elimination	2	0	LumB	PR	no	66
K677/2	5p8q9q10p16p	8q	elimination	2	1	LumB	SD	no	22
D390/2	5p8q10p	5p8q	elimination	2	1	LumB	SD	no	24
K187/1	no	no	no change	2	1	LumB	PR	no	28
M187/2	no	no	no change	2	1	LumB	PR	no	98
S667/1	no	no	no change	2	1	LumB	PR	no	26
D121/1	no	no	no change	2	0	LumB	PR	no	32
Sh332/1	no	no	no change	2	0	LumB	PR	no	71
V134/4	8q10p	8q10p	no change	2	0	LumB	PR	no	43
J134/2	5p8q16p	5p8q16p	no change	4	0	LumB	PR	no	115
Ch341/1	5p8q16p	5p8q16p	no change	3	3	LumB	PR	no	21
D256/2	5p8q9q10q	5p8q9q10q	no change	2	1	LumB	PR	Yes	19
P167/3	6p7q8q9p	6p7q8q9p	no change	4	1	TNBC	PR	no	63
N111/2	6p7q8q10p10q16p	6p7q8q10p10q16p	no change	2	2	TNBC	PR	no	32
B278/1	no	no	no change	1	1	LumB	SD	no	130
M289/3	no	no	no change	2	0	LumB	SD	no	73
K544/1	no	no	no change	2	0	TNBC	SD	no	38
G373/2	5p	5p	no change	2	1	TNBC	SD	no	98
J259/4	8q16p	8q16p	no change	2	1	LumB	SD	no	26
Ch233/3	5p7q8q10p16p	5p7q8q10p16p	no change	2	0	LumB	SD	no	41
G178/3	3q5p8q	3q5p8q	no change	2	0	LumB	SD	Yes	62
S156/1	5p7q16p18q19p	5p7q16p18q19p	no change	2	2	LumB	SD	Yes	24
P244/3	8q18q	6p8q18q	induction	2	0	LumB	PR	no	31
B156/5	16p	5p16p	induction	2	1	LumB	PR	Yes	25
Yu176/4	6p8q	6p8q13q	induction	2	3	LumB	PR	Yes	43
K743/3	8q9q13q	3q6p8q9q13q	induction	2	1	LumB	PR	Yes	12
L188/4	8q	8q10p	induction	2	3	LumB	SD	Yes	10
K299/2	8q16p	5p8q16p	induction	2	0	LumB	SD	Yes	105
R198/4	7q9p10p	5p7q9p10p	induction	4	3	LumB	SD	Yes	12
S232/2	6p9p10p	6p7q8q9p10p	induction	2	1	LumB	SD	Yes	20
Ch145/1	5p7q8q16p	5p7q10q8q16p	induction	2	0	LumB	SD	Yes	23
E106/2	8q	3q16p18q	induction	2	2	LumB	PD	Yes	47
S300/3	8q	5p7q8q13q19p	induction	3	3	TNBC	PD	Yes	17

Abbreviations: MFS, metastasis-free survival; LumB, luminal B breast cancer; TNBC, triple-negative breast cancer; PD, progressive disease; pCR, pathological complete response; PR, partial response; SD, stable disease.

Thirty-six patients had two or more amplifications at regions of stemness gene localization in their tumors before treatment. Elimination of clones with amplifications of stemness genes under NAC occurred in 53% of patients (19/36) (A126/3, B394/2, B386/2, V396/2, G265/5, D390/2, I133/4, K345/2, K467/2, K677/2, L234/2, L355/3, M366/2, M483/1, S454/3, S577/2, S702/2, Sh198/2, Ya134/1), and none of these patients developed metastases. In 10 of 36 patients, the number of clones with stemness gene amplifications did not change during NAC (V134/4, G178/3, D256/2, J134/2, J259/4, N111/2, P167/3, S156/1, Ch233/3, Ch341/1), and 30% (3 of 10) patients developed hematogenic metastases (G178/3, D256/2, S156/1). Seven patients manifested an induction in the number of clones with amplifications during NAC (K299/2, K743/3, P244/3, R198/4, S232/2, Ch145/1, Yu176/4), and 86% (6/7) of patients developed metastases (Table 1).

**Figure 1:**
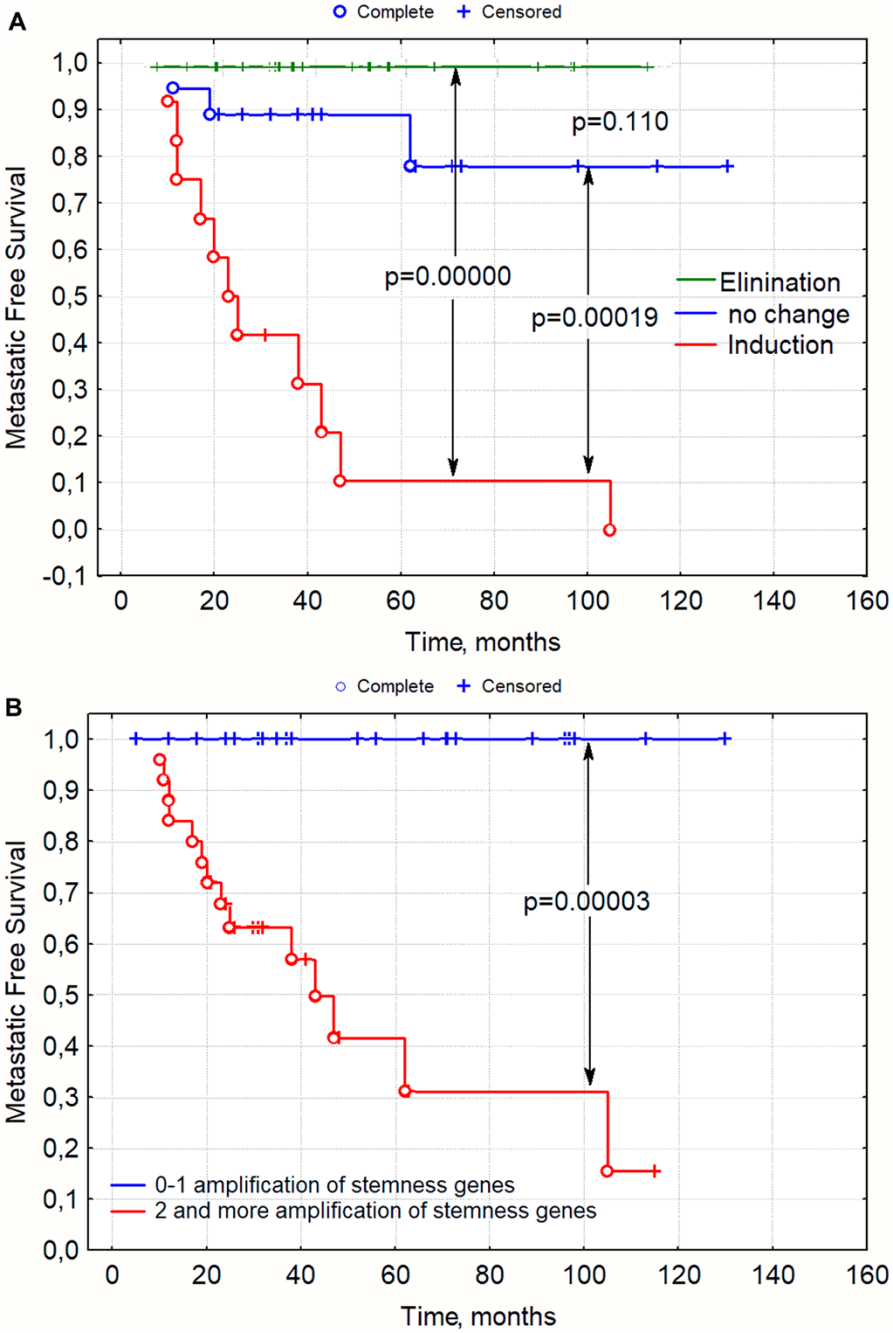
Metastasis-free survival rate in patients with breast cancer depending on changes in stemness gene amplifications during NAC (1a) and presence of amplifications in the residual tumor after NAC, (1b) *p*-value–log rank test. Note: On the abscissa: time after treatment in months, on the ordinate: metastasis-free survival; (**A**) Groups were formed in accordance with Table 1. Change during NAC. (**B**) Groups were formed in accordance with column 3, depending on the number of amplifications of stemness genes greater than or equal to 2 or less than 2.

If we estimate only the change in amplification of stemness genes in a tumor after NAC, all examined patients could be divided into three groups. The induction of new clones with amplifications at regions of stemness gene localization during NAC was associated with a very high rate of metastasis (91% - 10/11) (B156/5, E106/2, K299/2, K743/3, L188/4, R198/4, S232/2, S300/3, Ch145/1, Yu176/4); in the absence of changes in regions of stemness gene localization, the incidence of metastasis was low (16% - 3/19 - G178/3, D256/2, S156/1), and a 100% survival rate of all 20 patients was observed when the number of clones with stemness gene amplifications were eliminated under NAC (Table 1 and Figure 1A). Similar data were obtained when all the patients were divided into groups of those with two or more amplifications in the residual tumor and those with no or one amplification in the tumor after NAC (Figure 1B). The rate of metastasis in the group of patients with 0–1 amplifications was 0%, and with two or more amplifications it amounted to 54% (13/24) (Figure 1B). Eleven patients with two or more amplifications of stemness genes developed no metastases, but three patients underwent elimination of clones with amplifications, eight manifested no changes and the phenomenon of tumor «fading», and only one patient showed an increase.

Thus, we obtained data demonstrating an association of stemness gene amplifications with metastasis. The occurrence of amplifications at regions of stemness gene localization during NAC or the presence of two or more amplifications in the residual tumor was associated with a dramatically high rate of metastasis. In the absence of amplification or with one amplification in a tumor after NAC, none of the 26 patients developed metastases.

We have previously shown that ten patients (having the luminal B subtype with a tumor size less than 1 cm (T1), which is a favorable sign, and did not perform NAC) had visceral metastases at various times after surgery (from 3 to 120 months). In 9 of 10 (90%) of these patients, there were two or more amplifications of the stemness genes in the primary tumor, which, in our opinion, predetermined an unfavorable outcome in these patients [23].

Thus, the presence of 2 or more amplifications of stemness genes is linked to metastasis. The induction of new amplifications in the tumor under the influence of chemotherapy led to a dramatically high frequency of metastasis (91% of cases). The tumor did not metastasize if there was only one or no amplifications in the residual tumor.

NAC for patients with stemness gene amplifications showed that patients have a chance to eliminate them prior to NAC, after which a 100% survival rate was observed. NAC for patients without stemness gene amplifications before treatment and especially with one amplification was very likely (67%) to lead to stimulation of their occurrence and metastasis.

### Association of stemness gene expression in breast tumors with metastasis

In the next stage, we studied the expression of 13 stemness genes in 98 patients (*TERT*, *OCT3*, *SMO*, *MYC*, *SNAI2*, *TGFBR1*, *KLF4*, *BMI1*, *VIM*, *FLT3*, *SMAD2*, *KLF1*, and *TGFβ1)* with breast tumors before treatment and after NAC, and we compared the expression levels of these genes in patients with metastases (32 of 98) and without metastases (66 of 98). We isolated RNA from biopsies before treatment and surgical tumor material after NAC, and gene expression was evaluated by qPCR. We wanted to show that the expression of stemness genes in the tumor was increased in patients with metastases.

It has been shown that patients with metastases manifest a statistically relevant high level of expression of two stemness genes (*MYC*, *BMI1*) in tumors before treatment and three more at the trend level. After NAC, patients with hematogenic metastases manifested a statistically relevant high expression level of 7 stemness genes (*OCT3*, *SMO*, *MYC*, *SNAI2*, *VIM*, *TERT, TGFB1*) in their residual tumors and another three at the trend level (Table 2). An increase in expression of stemness genes in a residual tumor in patients with metastases under the influence of NAC showed an abnormal up-regulation or ectopic expression of stemness genes.

**Table 2:** Expression of stemness genes in biopsies of breast tumors

Genes		No metastasis (*n* = 66)	Yes metastasis (*n* = 32)	*p*-value		No metastasis (*n* = 66)	Yes metastasis (*n* = 32)	*p*-value
*OCT3*	Before NAC	5.98 ± 2.89	12.31 ± 8.15	0.366	After NAC	**10.63 ± 5.01**	**64.12 ± 35.68**	**0.040**
*SMO*		0.57 ± 0.09	0.80 ± 0.14	0.167		**0.80 ± 0.15**	**1.53 ± 0.43**	**0.047**
*MYC*		**0.41 ± 0.04**	**0.72 ± 0.16**	**0.012**		**0.63 ± 0.10**	**1.94 ± 0.78**	**0.020**
*SNAI2*		0.49 ± 0.08	0.73 ± 0.16	0.144		**0.99 ± 0.15**	**1.77 ± 0.43**	**0.035**
*KLF4*		0.76 ± 0.12	1.07 ± 0.28	0.224		2.21 ± 0.44	3.82 ± 2.47	0.378
*BMI1*		**2.14 ± 0.37**	**4.07 ± 0.75**	**0.011**		34.63 ± 31.93	8.06 ± 3.55	0.565
*VIM*		0.30 ± 0.04	0.41 ± 0.06	0.137		**0.69 ± 0.11**	**1.56 ± 0.59**	**0.049**
*FLT3*		2.49 ± 0.50	2.18 ± 0.85	0.746		2.59 ± 0.76	4.52 ± 1.60	0.218
*SMAD2*		0.19 ± 0.03	0.37 ± 0.27	0.338		0.35 ± 0.06	0.32 ± 0.12	0.778
*KLF1*		0.29 ± 0.08	0.31 ± 0.10	0.864		0.44 ± 0.22	0.53 ± 0.33	0.811
*TERT*		0.43 ± 0.10	1.26 ± 0.61	0.061		**0.35 ± 0.07**	**1.53 ± 0.68**	**0.016**
*TGFB1*		1.00 ± 0.14	1.63 ± 0.38	0.062		**1.61 ± 0.26**	**3.08 ± 0.95**	**0.047**
*TGFBR1*		0.85 ± 0.13	1.02 ± 0.19	0.458		1.22 ± 0.17	1.99 ± 0.79	0.201

### The significance of stemness gene amplifications for non-CSC to CSC plasticity

In the next stage, we decided to develop an *in vitro* model in which tumor cells would be able to differentiate, reproduce and form mammospheres in the presence of stemness gene amplification. In the absence of stemness gene amplification, no process of dedifferentiation would occur, proliferative activity would be high, and mammospheres would form.

**Figure 2:**
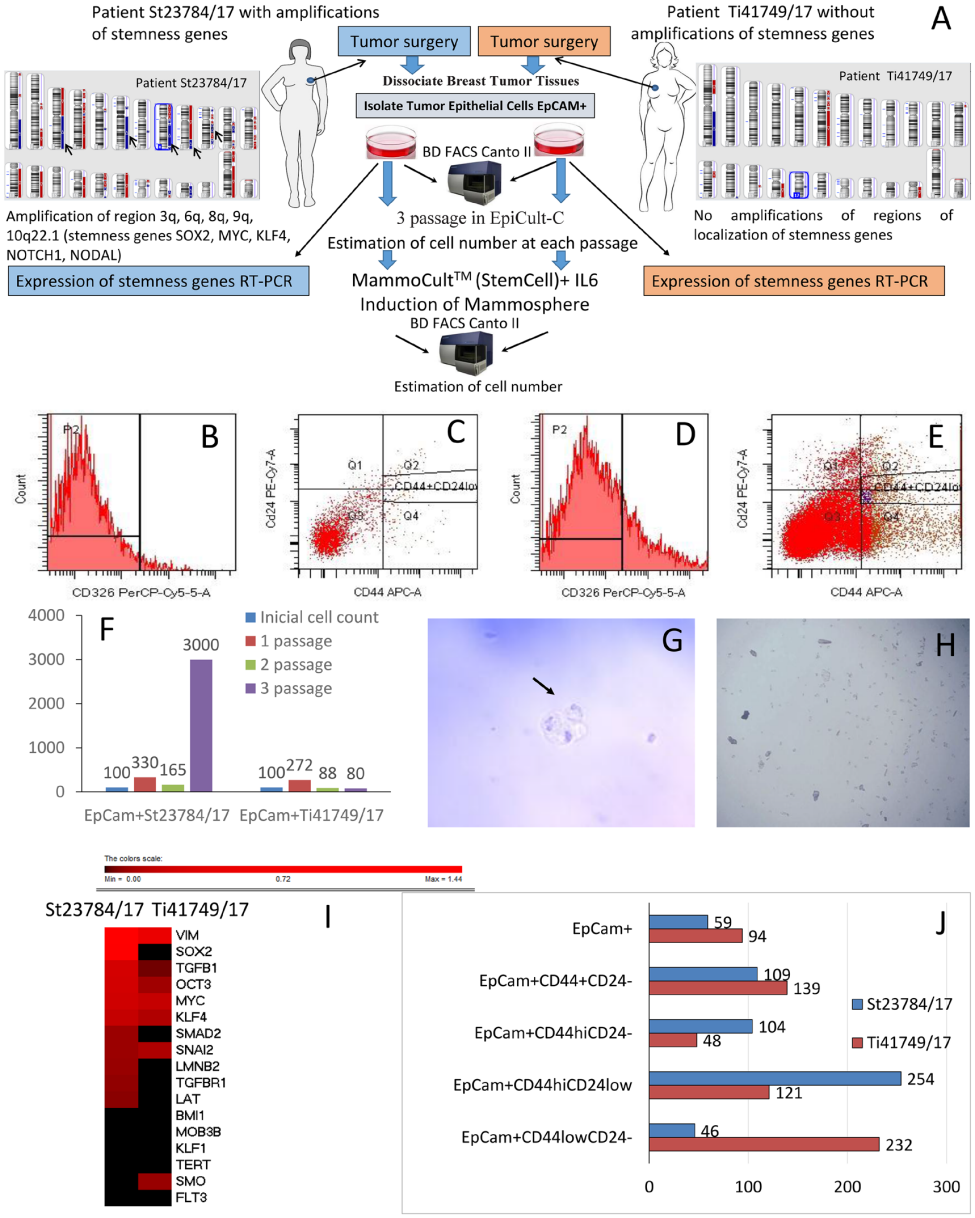
The significance of stemness gene amplifications for stem-like cancer cell activity. (**A**) The experimental design and the CNA genetic landscape of tumors in patients St23784/17 and Ti41749/17, showing that St23784/17 had amplifications at the 3q, 6q, 8q, 9q, and 10q22.1 regions of stemness gene localization (the following genes were amplified: *SOX2*, *MYC*, *KLF4*, *NOTCH1*, *NODAL*), and Ti41749/17 had no stemness gene amplifications. (**B**–**E**) Analysis of the quantitative and qualitative expression of cells obtained from tumor tissue of breast cancer patients. The cells were analyzed by flow cytometry using a FACSCanto II (Becton Dickinson, USA) and FACS Diva software, using CD326 (PerCP-Cy5.5), CD24 (PE-Cy 7), and CD44 (APC) (BD Biosciences, USA) antibodies. (B) Histogram of the IgG1 PerCP Cy5.5 isotope control; (C) isotype control dot-plot for IgG2a PE-Cy 7/IgG2b APC; (D) phenotypic confirmation and qualitative analysis of CD326 (PerCP-Cy5.5) expression in the histogram; (E) phenotypic confirmation and qualitative analysis of CD326^+^CD44^+^CD24^-\low^ expression in the dot-plot. (**F**) The dynamics of the EpCam+ tumor cell mass increase (percentage of the initial number of cells in culture). (**G**) Nonadherent mammospheres of EpCam^+^ cancer cell cultures (St23784/17 patient). Mammosphere formation within 10 days. Scale bar, 200 μm. (**H**) EpCam^+^ cells without mammosphere formation (patient Ti41749/17). (**I**) Thermal map of stemness gene expression in the environment enriched with EpCam+ tumor cells from the patients St23784/17 and Ti41749/17. (**J**) The content of tumor stem cells in the EpCam^+^ cell enriched environment after a cycle of cultivation and IL-6 treatment (% of the initial cell content in each population).

During surgery, we collected tumor material from two breast cancer patients who had not undergone NAC to study the significance of stemness gene amplifications in relation to the activity of stem-like cancer cells. One patient (St23784/17) had amplifications in the 3q, 6q, 8q, 9q, and 10q22.1 regions of stemness gene localization in her tumor (the following genes were amplified: *SOX2*, *MYC*, *KLF4*, *NOTCH1*, *NODAL*). There were no stemness gene amplifications in the tumor from patient Ti41749/17. Figure 2A presents the design of the experiment and the CNA-landscape of the patient tumors, confirming the presence of stemness gene amplification in St23784/17 and absence of stemness gene amplifications in Ti41749/17.

The content of cancer stem cells (CSCs) was significantly higher in the St23784/17 than in Ti41749/17. The differences in cells with the EpCam^+^CD44^+^CD24^–^ and EpCam^+^CD44^hi^CD24^–^ phenotypes were especially apparent. The content of EpCam^+^CD44^+^CD24^–^ and EpCam^+^CD44^hi^CD24^–^ subpopulations in patient St23784/17 was 1.7 and 2.1 times higher than in patient Ti41749/17, respectively (Table 3).

**Table 3:** Stem cell populations (CD326+ fraction) in two breast tumors with (ST23784/17) and without (Ti41749/17) amplification of stemness genes

Cells	Phenotype	Tumor of patient St23784/17	Tumor of patient Ti41749/17
Total epithelial cell population EpCam+	EpCam^+^	18,03	5,10
Epithelial cancer stem cell population	EpCam^+^CD44^+^CD24^–^	51,28	30,34
CD44 high expression and CD24^–^ CSC population	EpCam ^+^CD44^hi^CD24^–^	37,88	18,14
CD44 high expression and CD24low expression CSC population	EpCam ^+^CD44^hi^CD24^low^	2,71	3,84
CD44 low expression and CD24^–^ population	EpCam ^+^CD44^low^CD24^–^	6,83	4,85

Note: The content of the tumor stem cell populations in two patients after immunomagnetic separation (% of stained mononuclear cells).

We studied the expression of stemness genes in the EpCam^+^ cells derived from the breast tumor of patient St23784/17 with stemness gene amplifications and from the tumor of patient Ti41749/17 with no stemness gene amplifications. Increased expression of 10/16 stemness genes studied (*SOX2*, *OCT3*, *MYC*, *TGFBR1*, *KLF4*, *TGFB1*, *LAT*, *LMNB2*, *SMAD2*, *VIM*) was also observed in the EpCam^+^ tumor cells extracted from the tumor of patient St23784/17 compared with the EpCam^+^ tumor cells from patient Ti41749/17 (Figure 2I).

EpCam^+^ cells derived from the breast tumor of patient Ti41749/17 were cultivated in a Human EpiCult-C environment. The EpCam^+^ cell culture survived 3 passages. Microscopic examination revealed that most of the cells in culture were small and rounded; additionally, the culture contained large cells with an epithelial morphology. After the first passage, we observed a significant increase in cell mass; the number of EpCam^+^ cells was three times higher than the initial level (Figure 2F).

In the specialized Human EpiCult-C environment, the increase in EpCam^+^ breast tumor cells of breast cancer patient St23784/17 differed from the culture of EpCam^+^ cells of patient Ti41749/17. More than a 3-fold increase in cell mass was observed after the first passage, and the third passage showed a sharp increase of more than 30-fold in cell mass (Figure 2F).

We estimated individual populations of cells and compared them to the content after magnetic separation in the general fraction of the third-passage EpCam^+^ cells from patient Ti41749/17 with no amplifications after cultivation in the MammoCult specialized environment. As shown in Figure 2J, the number of cells with the EpCam^+^CD44^low^CD24^–^ phenotype was significantly higher (2.5 times) after cultivation in the MammoCult specialized environment (with IL-6) than after magnetic separation in the general fraction of the third-passage EpCam+ (without IL-6). We did not observe the formation of mammospheres from the EpCam-positive cell population of third-passage EpCam+ cells in the specialized MammoCult environment, even after the addition of IL-6 (Figure 2H).

The general fraction of third-passage EpCam^+^ cells from patient St23784/17 with amplifications after incubation with IL-6 showed an increase in the number of cells with the EpCam^+^CD44^hi^CD24^–^ phenotype, a significant increase in the number of EpCam^+^CD44^hi^CD24^low^ and a sharp decrease in the number of EpCam^+^CD44^low^CD24^–^ phenotype cells (Figure 2J). We observed no colony formation from third-passage EpCam^+^ cells from patient St23784/17 in the MammoCult environment. Ten days after the addition of IL-6, mammospheres were identified (Figure 2G).

## DISCUSSION

A retrospective study of changes in the CNA-genetic landscape of breast tumor during NAC in 50 patients showed that the presence of 2 or more stemness gene amplifications in a residual tumor was associated with metastasis. If NAC stimulated the formation of new stemness gene amplifications, then the tumor metastasized in 91% of cases. NAC is advisable in patients with amplifications, since 53% of cases showed the possibility of elimination of tumor clones with stemness gene amplifications. Elimination of clones with stemness gene amplifications resulted in 100% metastasis-free survival (Table 1 and Figure 1).

Many studies have shown that chemotherapy can eliminate tumor clones and leads to increased metastasis-free survival. It is well known that when conducting NAC for breast cancer patients, a complete morphological regression is achieved (in such cases, the elimination of clones with amplifications is practically guaranteed [24, 25].

Conversely, an increasing number of studies have stated that in some patients, chemotherapy and targeted therapy can stimulate the formation of new mutations, which leads to resistance formation and tumor progression. Findlay et al. (2016) examined 30 esophageal adenocarcinoma samples before treatment and after two courses of pre-operative chemotherapy under the oxaliplatin-fluorouracil scheme. Patients who did not respond to chemotherapy developed mutations of *TP53* in their tumors after treatment (none in the initial samples), which correlated with adverse outcomes (≈ 20% of patients). Neoadjuvant chemotherapy in some cases stimulated breast cancer metastasis through micro-anatomical structures called the tumor microenvironment of metastasis (TMEM) [26]. Additionally, results in some patients showed that chemotherapy could contribute to the stages of the metastasis process: stimulate epithelial-mesenchymal transition (EMT) in tumor cells [7], stimulate invasion, intravasation, inflammation [27], circulating tumor cell (CTC) release into the blood [6], and facilitate metastasis into organs by increasing the adhesion of tumor cells to the blood vessel endothelium through increased expression of VEGFR1 [8].

Our studies showed that in metastasis, the residual tumor expressed 7/13 stemness genes, demonstrating a statistically relevant high level (Table 3) as compared to residual tumors of patients without metastases. Increased expression of 10/16 of the stemness genes studied was also observed in EpCam^+^ tumor cells extracted from the tumor of patient St23784/17 with amplifications at 3q, 6q, 8q, 9q, 10q22.1 regions of localization stemness genes compared with EpCam^+^ tumor cells from patient Ti41749/17 with no stemness gene amplifications. The observed ectopic expression of stemness genes in patient St23784/17 led to high proliferative activity of EpCam+ tumor cells and induction of mammospheres due to the EpCam+ tumor stem cell fraction under the influence of IL-6. In contrast, in the absence of stemness gene amplifications, the proliferation of EpCam+ tumor cells from patient Ti41749/17 was reduced, and EpCam+ tumor cells from patient Ti41749/17 did not form mammospheres, even under the influence of IL-6.

Initially, the patient with stemness gene amplifications had a high concentration of EpCam^+^CD44^+^CD24^–^ tumor stem cells – more than 50%, while patient Ti41749/17 had a level slightly above 30% and a two-fold higher concentration of EpCam^+^CD44^hi^CD24^–^ cells (37% compared with 18%). Even after incubation with IL-6, the concentration of EpCam^+^CD44^+^CD24^–^ tumor stem cells was 1.33 times higher in patient St23784/17 than patient Ti41749/17. Moreover, under the influence of IL-6, the concentration of EpCam^+^CD44^hi^CD24^low^ tumor stem cells increased dramatically (2.5 times), and the EpCam^+^CD44^low^CD24^–^ concentration decreased two-fold (Figure 2). As described by Cristine Chaffer et al. (2013) [4], this phenomenon may indicate the launch of a dedifferentiation program in EpCam^+^CD44^low^CD24^–^ tumor cells with amplifications from the St23784/17 patient, or CD44^low^CD24^–^ в CD44^hi^CD24^low^ non-CSC to CSC plasticity. Tumor cells with no stemness gene amplifications from patient Ti41749/17 displayed a diametrically opposite process. After incubation with IL-6, the total concentration of the EpCam^+^CD44^hi^CD24^–^ and EpCam^+^CD44^hi^CD24^low^ cells decreased, and the concentration of the EpCam^+^CD44^low^CD24^–^ tumor cells drastically increased (2.3 times), i. e., although the tumor stem cells were beginning to proliferate in patient Ti41749/17, they underwent differentiation, gradually reducing their stem potential. Thus, the induction of stem properties and non-CSC to CSC plasticity - dedifferentiation of CD44^low^ to CD44^hi^ in CD326^+^ tumor cells, occurred only with stemness gene amplifications. These data are limited to the study of only two primary cultures of tumor cells. In future works, it will be necessary to increase the number of primary cultures. In addition, using standard tumor cultures and CRISPR/CAS9, non-CSC to CSC plasticity can be modeled and the value of stemness gene amplifications shown.

Currently, numerous studies have shown the induction of stem properties in tumor cells under the influence of forced expression of stemness genes. Herreros-Villanueva et al. (2013) showed *SOX2* ectopic expression in 19.3% of human pancreatic tumors, which was not present in normal pancreatic cells. *SOX2* knockdown in pancreatic cancer cells leads to an inhibition of cell growth, while *SOX2* transfection promotes cell entry into S-phase and proliferation, and it also increases the expression levels of CSC pancreatic *ALDH1*, ESA, and CD44 markers [14]. Transfection of *SOX2* into hepatocellular carcinoma cells results in the expression of CD133 and *OST3* stem cell markers and mammosphere formation. *SOX2* knockdown reduces the expression of stem cell markers and reverses the formation of mammospheres [12]. In 2012, Kumar and colleagues showed that transmembrane delivery of Oct3 protein contributes to the dedifferentiation of melanoma cells into stem-like cancer cells. RNAi-mediated Oct3 knockdown in dedifferentiated cells leads to a decrease in the CSC phenotypes [11]. A review by Simple and colleagues (2015) and the original article by Suvà et al. (2014) provided a number of additional examples of stem property activation in tumor cells under the action of stemness genes [9, 28]. It has recently been shown that *OCT3*, *SOX2*, and *NANOG* are specifically hypomethylated in clustered CTCs, and that pharmacological dissociation of CTC clusters reverts their methylation profile and suppresses metastasis [29].

**Figure 3:**
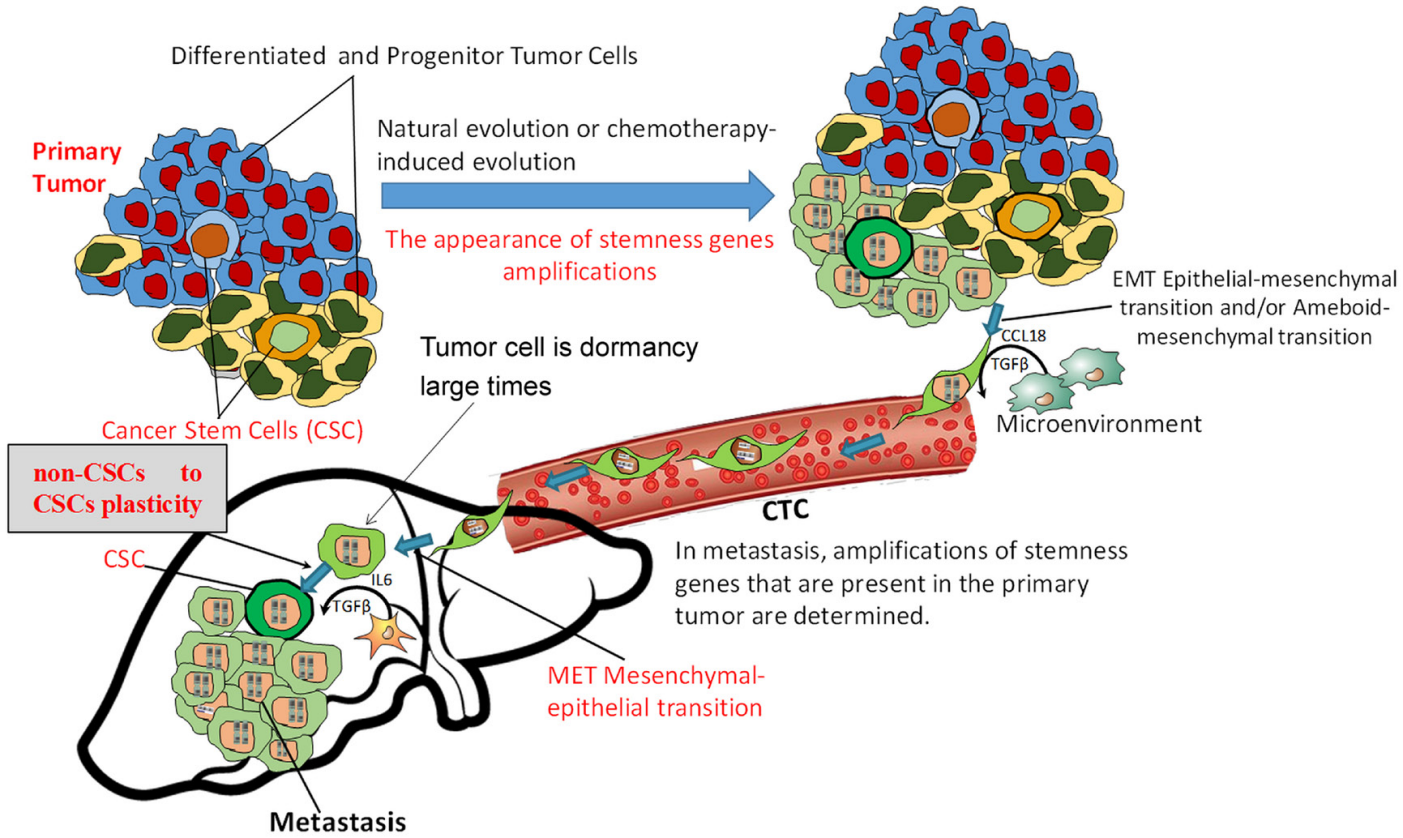
Metastasis scheme indicating the place of non-CSC to CSC plasticity in the metastatic cascade. The tumor acquires the ability to metastasize due to ectopic expression of stemness genes, which is primarily due to the amplification of their loci. As a result of ectopic expression of stemness genes, the development of non-CSC to CSC plasticity and the formation of cancer stem cells can occur. Non-CSCs enter the secondary organ, become CSCs, and metastatic colony development ensues. In the absence of ectopic expression of stemness genes, the tumor will produce CTCs, but a lack of non-CSC to CSC plasticity and development of a stem phenotype in the internal organs will not allow such tumors to metastasize.

Thus, the data obtained by our group mechanistically shows that the presence of stemness gene amplifications in tumor cells led to ectopic expression of an entire cluster of stemness genes and boosted the proliferative activity of EpCam^+^ tumor cells, increased the concentration of EpCam^+^CD44^+^CD24^–^ cancer stem cells, determined the EpCam^+^CD44^low^CD24^–^ to EpCam^+^CD44^hi^CD24^low^ plasticity and was able to induce mammosphere formation. Apparently, the EpCam^+^CD44^low^CD24^–^ phenotype represents a subpopulation of progenitor tumor cells that are capable of non-CSC to CSC plasticity, and further studies are needed to assess their importance in metastasis based on clinical data. From the results of this work, we revealed certain changes in the known metastatic cascade and determined the place of non-CSC to CSC plasticity therein (Figure 3). At a certain stage of carcinogenesis, under the influence of internal factors and the microenvironment or in the process of chemo-induced clonal evolution [30], the tumor acquires the ability to metastasize. The reason for this ability is the ectopic expression of stemness genes, which is primarily due to the amplification of their loci, although other mechanisms (epigenetic, mutations, microRNA, etc.) should not be disregarded. As a result of the ectopic expression of stemness genes, the activity of cancer stem cells and stem-like cancer cells significantly increases; such cells can be formed from differentiated tumor cells as a result of non-CSC to CSC plasticity.

We think that a sharp increase in stem activity of tumor cells and a high probability of developing non-CSC to CSC plasticity in secondary organs in cases of stemness gene amplifications are the cause of metastatic colony formation. In the absence of ectopic expression of stemness genes, the tumor will produce CTCs, but their low proliferative activity, lack of non-CSC to CSC plasticity ability and formation of a stem phenotype in the internal organs will not allow such tumors to metastasize, even if all other features (invasion, intravasation, circulation in the blood flow, extravasation, adaptation) are normal. Thus, many researchers note the low prognostic significance of the presence and number of CTCs.

## MATERIALS AND METHODS

### Patients and therapy

The study included 98 patients with IIA - IIIB (*T*1–4*N*0–3*M*0) breast cancer of the luminal B and triple-negative molecular subtypes aged from 28 to 66 years (average age of 47.4 ± 0.8 years) without BRCA1 and BRCA2 germinal mutations who received treatment at the Cancer Research Institute of Tomsk NRMC (Tomsk, Russia) from 2008–2014. All patients had their tumor CNA-genetic landscape studied before treatment using a CytoScan HD Array (Affymetrix, USA), and 50 patients had their tumor CNA-genetic landscape alterations studied after neoadjuvant chemotherapy (NAC).

All patients received 6–8 cycles of systemic anthracycline-based NAC (AC), anthracycline and taxane-based NAC (ACT or AT) or taxotere in the monoregimen. Physical examination was performed before NAC and before surgery to determine the clinical response. Imaging of the primary breast lesion was performed by mammography and ultrasonography, and clinical and imaging responses were categorized as follows: clinical complete response (CR), partial response (PR), stable disease (SD), progressive disease (PD). An immunohistochemical study was conducted to determine the molecular subtype of the tumor before treatment. The luminal B subtype of breast cancer was defined as ER^+^, PR^+^ or -, Ki67 > 30%, and all patients with the luminal B subtype were HER2-negative. Some patients showed no expression of ER, PR, and HER2, and they were classified as a triple-negative subtype. We conducted a morphological study to assess the pathological response to NAC by RCB after treatment, and at RCB-0, we discussed the pathologic complete response (pCR). Patients underwent radiation therapy and/or hormone therapy after surgery. Hormone therapy was prescribed to all patients with the luminal B subtype. Radiation therapy was prescribed in the case of lymphatic metastasis. The main clinical and morphological data of the patients are presented in Table 4.

**Table 4:** Clinical and morphological parameters of the examined breast cancer patients

Clinical and morphological parameters		Number of patients (%)
Age (years)	≤ 45	37 (37.8%)
	> 45	61 (62.2%)
Menstrual status	Premenopause	53 (54.1%)
	Postmenopause	45 (45.9%)
Histological type	Invasive ductal carcinoma	93 (94.9%)
	Other types	5 (5.1%)
Tumor size	T_1_	9 (9.2%)
	T_2_	78 (79.6%)
	T_3_	5 (5.1%)
	T_4_	6 (6.1%)
Lymphogenous metastasis	N_0_	40 (40.8%)
	N_1_	46 (46.9%)
	N_2_	5 (5.1%)
	N_3_	7 (7.2%)
Molecular subtype	Luminal B HER2-	82 (83.7%)
	Triple negative	16 (16.3%)
Histological form	Unicentric	66 (67.3%)
	Multicentric	32 (32.7%)
NAC scheme	AC	57 (58.2%)
	Taxotere	23 (23.5%)
	AT/ACT	18 (18.3%)
Clinical Response to NAC	Progressive Disease (PD)	2 (2.0%)
	Stable Disease (SD)	26 (26.5%)
	Partial Regression (PR)	59 (60.2%)
	Complete Regression (CR)	9 (9.2%)
Distant metastasis	Yes metastasis	32 (32.7%)
	No metastasis	66 (67.3%)

Fresh breast cancer tissues were obtained during the initial diagnostic biopsy before NAC and during the course of tumor resection after NAC. The obtained tissue samples were stored in RNALater solution (Ambion, USA) within 24 hours at +4°C and then at –80°C per the manufacturer’s instructions until further use. Histological diagnosis was confirmed for all samples.

### DNA isolation

DNA was extracted from samples of pre- and post-NAC tumor tissues using the QIAamp DNA mini Kit (Qiagen, Germany #51304).

### Microarray analysis

To study CNAs of breast tumors, microarray analysis was performed using a high-density microarray platform, Affymetrix (USA) CytoScan™ HD Array, (http://www.affymetrix.com/esearch/search.jsp?pd=prod520004&N=4294967292). Procedures for sample preparation, hybridization and scanning were performed in accordance with the manufacturer’s protocol using the Affymetrix GeneChip^®^ Scanner 3000 7G system (Affymetrix, USA). The Chromosome Analysis Suite 4.0 software (Affymetrix, USA), which is specifically devised for analyzing microarray results from the CytoScan™ HD Array, was used. Unbalanced chromosomal aberrations (deletions and amplifications, or loss and gain) were detected in all chromosomal regions

### RNA isolation

We obtained RNA from biopsy tumor material before treatment and from surgical tumor material after NAC, as well as from tumor cell cultures. We isolated RNA using RNeasy Mini Kit Plus (Qiagen, Germany), which contains DNase to degrade DNA.

### Evaluation of stemness gene expression

The level of stemness gene expression was assessed by quantitative real-time reverse transcription PCR (qPCR) via TaqMan technology on a RotorGene-6000 amplifier (Corbett Research, Australia). To obtain cDNA, a reverse transcription reaction was performed on the RNA array using the RevertAid™ Kit (Thermo Scientific, USA) with random hexanucleotide primers according to the kit instructions.

PCR was performed in three replicates [31]. Primers and probes (FAM-BHQ1) were chosen using Vector NTI Advance 11.5 software and the NCBI database (http://www.ncbi.nlm.nih.gov/nuccore) (Supplementary Table 2). The housekeeping gene of the *GAPDH* enzyme (glyceraldehydes-3-phosphate dehydrogenase) and the *ACTB* (Actin-β) gene were used as reference genes. The expression levels of each target gene were normalized to *GAPDH* and *ACTB* expression. For tumor tissue, the relative expression of stemness genes was evaluated using the Pfaffl method [32]. We used the difference in the expression of *TERT*, *OCT3*, *SMO*, *MYC*, *SNAI2*, *TGFBR1*, *KLF4*, *BMI1*, *VIM*, *FLT3*, *SMAD2*, *KLF1*, and *TGFβ1* relative to the expression of *GAPDH* and *ACTB* genes and expression in normal breast tissue. For cell cultures, the relative expression of stemness genes (*SOX2, OCT3; SMO, MYC, SNAI2, MOB3B, TGFBR1, KLF4, BMI1, VIM, FLT3, LAT, SMAD2, LMNB2, KLF1*, and *TGFβ1*) was estimated relative to the expression of *GAPDH* and *ACTB* from normal breast tissue.

### Flow cytometry

The following antibodies were used for cell surface staining of mononuclear cells derived from tumors of patients and cell lines: PE-Cy7-conjugated anti-CD24, APC-conjugated anti-CD44, and PerCP-Cy5-conjugated anti-CD326 (EpCam) (BD Biosciences, USA). Cells were stained at a concentration of 1 × 10^6^ cells/ml in FACS buffer (PBS containing 0.1% BSA) for 30 minutes on ice in the dark, after which the cells were washed twice in FACS buffer. The following isotype control groups were used: PerCPCy5.5 IgG1, APC IgG2b, and PE-Cy7 IgG2a. Stained cells were analyzed using a FACSCanto II (Becton Dickinson) with the FACS Diva software program.

### Dissociation of breast cancer cells

Breast cancer was dissociated according to the technical protocol supplied by StemCell Technologies (Canada). These procedures have been optimized to dissociate human mammary tissue or breast cancer tissue to a single-cell suspension for use in cell separation, flow cytometry or progenitor cell assays. Human breast tumor samples were dissociated overnight with collagenase/hyaluronidase enzymes (StemCell Technologies, Catalog #079128). The human mammary organoids, obtained through differential centrifugation, can then be dissociated into a single cell suspension using trypsin/EDTA (StemCell Technologies, Catalog #07901), dispase (StemCell, Catalog #07913) and DNase (StemCell Technologies, Catalog #07900) enzymes.

### Isolation of breast cancer cells

The EpCam^+^ cell fraction was isolated using the EasySep™ Human EpCam Positive Selection Kit (StemCell Technologies, Catalog #18356). Cells were incubated for 20 minutes on ice with an EasySep™ Human EpCam Positive Cell Selection Cocktail, followed by an additional 15 minutes with magnetic nanoparticles. For EasySep™ Cell Separation, the labeled cell suspension was placed in the EasySep™ magnet for 5 minutes, and the cells that were not magnetically labeled were poured away. The labeled cells were resuspended, and the separation was repeated a total of 6 times. The EasySep™ Cell Separation was evaluated by flow cytometry.

### *In vitro* colony-forming assays (culture of human mammary epithelial cells and mammospheres or tumorspheres)

Separation-sorted human breast cancer cell concentrations were determined using the cell counter and then seeded at densities of 5 × 10^5^ cells/mL in low adherence 6-well plates. The cultures were maintained in Human EpiCult-C (StemCell Technologies, Catalog #05630) supplemented with 5% FBS (StemCell Technologies, Catalog #07904) for 24 hours, and then the medium was replaced with serum-free conditions and maintained for an additional 10 days. At the end of the assays, the colonies were enumerated under a microscope. After 10 days, the medium was removed and the plates gently rinsed with PBS. The culture cells were then counted. The procedure was repeated three times. Further, the sorted cells were seeded and cultured in the presence MammoCult™ (StemCell Technologies, Catalog #05620) supplemented with 0.48 μg/mL freshly dissolved hydrocortisone (StemCell Technologies, Catalog #07904) and 4 μg/mL heparin (StemCell Technologies, Catalog #07980) and IL-6 (Sigma, Catalog # SRP3096) to induce greater numbers of mammospheres and tumorspheres, and the cultures were maintained for an additional 7 to 10 days. At the end of the culture period, the colonies were enumerated under a microscope.

At the end of the assay, the cells were assessed by flow cytometry and image processing of each well with Cytation™ 3.

### Statistical analysis

The presence of structural CNA in breast tumor tissue was assessed using Chromosome Analysis Suite 3.1 software. Statistical analyses were performed using STATISTICA 8.0 software (StatSoft, Tulsa, OK, USA). The arithmetic mean value and standard error were calculated for each sample group, and the Mann-Whitney *U*-test was applied to identify the link between the expression levels of stemness genes in breast tumors. A two-sided *p*-value was calculated using Fisher’s exact test (http://vassarstats.net/odds2×2.html). Metastasis-free survival was calculated using the Kaplan-Meier method, and differences among patient groups were evaluated using the log-rank test.

## CONCLUSIONS

The data obtained in the course of this study show that the presence of amplifications at chromosomal regions of stemness gene localization in the tumor is associated with the ability to undergo metastasis and the possibility of developing non-CSC to CSC plasticity with active proliferation and mammosphere formation.

The development of medication aimed at the inhibition of non-CSC to CSC plasticity could become a new line of tumor treatment. The obtained results showing a significant increase in metastasis-free survival in patients without amplifications of stemness genes or with elimination of amplifications during NAC, support the continuation of research in this direction.

## SUPPLEMENTARY MATERIALS






