# Supplementary material for: Amplifications of stemness genes and the capacity of breast tumors for metastasis
Source: Oncotarget. 2020 May 26;11(21):1988–2001. doi: 10.18632/oncotarget.27608 (PMC7260118; doi:10.18632/oncotarget.27608)
Supplement: Supplementary file 1 [file oncotarget-11-1988-s001.pdf]

Amplifications of stemness genes and the capacity of breast tumors for metastasis

SUPPLEMENTARY MATERIALS

REFERENCES

1. Tam WL, Ng HH. Sox2: masterminding the root of cancer. Cancer Cell. 2014; 26:3–5. https://doi.org/10.1016/j.ccr.2014.06.024. [PubMed]
2. Sarkar A, Hochedlinger K. The sox family of transcription factors: versatile regulators of stem and progenitor cell fate. Cell stem cell. 2013; 12:15–30. https://doi.org/10.1016/j.stem.2012.12.007. [PubMed]
3. Monk M, Hitchins M, Hawes S. Differential expression of the embryo/cancer gene ECS A (DPPA 2), the cancer/testis gene BORIS and the pluripotency structural gene OCT 4, in human preimplantation development. Mol Hum Reprod. 2008; 14:347–55. https://doi.org/10.1093/molehr/gan025. [PubMed]
4. Chakravarthy H, Boer B, Desler M, Mallanna SK, McKeithan TW, Rizzino A. Identification of DPPA4 and other genes as putative Sox2: Oct-3/4 target genes using a combination of in silico analysis and transcription-based assays. J Cell Physiol. 2008; 216:651–62. https://doi.org/10.1002/jcp.21440. [PubMed]
5. Liu CW, Li CH, Yi-Jen P, Cheng YW, Chen HW, Liao PL, Kang JJ, Yeng MH. Snail regulates Nanog status during the epithelial–mesenchymal transition via the Smad1/Akt/GSK3β signaling pathway in non-small-cell lung cancer. Oncotarget. 2014; 5:3880–3894. https://doi.org/10.18632/oncotarget.2006. [PubMed]
6. Vinagre J, Pinto V, Celestino R, Reis M, Pópulo H, Boaventura P, Melo M, Catarino T, Lima J, Lopes JM. Telomerase promoter mutations in cancer: an emerging molecular biomarker? Virchows Arch. 2014; 465:119–33. https://doi.org/10.1007/s00428-014-1608-4. [PubMed]
7. Shen R, Jia R, Liu W, Lin Q, Hai Y, He Z. The function and regulation of BMP6 in various kinds of stem cells. Curr Pharm Des. 2015; 21:3634–43. https://doi.org/10.2174/1381612821666150710150218. [PubMed]
8. Cho Y, Kang HG, Kim SJ, Lee S, Jee S, Ahn SG, Kang MJ, Song JS, Chung JY, Eugene CY. Post-translational modification of OCT4 in breast cancer tumorigenesis. Cell Death Differ. 2018; 25:1781–1795. https://doi.org/10.1038/s41418-018-0079-6. [PubMed]
9. Zhang J, Liang Q, Lei Y, Yao M, Li L, Gao X, Feng J, Zhang Y, Gao H, Liu DX. SOX4 induces epithelial–mesenchymal transition and contributes to breast cancer progression. Cancer Res. 2012; 72:4597–608. https://doi.org/10.1158/0008-5472.CAN-12-1045. [PubMed]
10. Soriano JV, Uyttendaele H, Kitajewski J, Montesano R. Expression of an activated Notch4 (int-3) oncoprotein disrupts morphogenesis and induces an invasive phenotype in mammary epithelial cells *in vitro*. Int J Cancer. 2000; 86:652–9. https://doi.org/10.1002/(sici)1097-0215(20000601)86:5<652::aid-ijc8>3.0.co;2-v. [PubMed]
11. Kim SH, Singh SV. The role of polycomb group protein Bmi-1 and Notch4 in breast cancer stem cell inhibition by benzyl isothiocyanate. Breast Cancer Res Treat. 2015; 149:681–92. https://doi.org/10.1007/s10549-015-3279-5. [PubMed]
12. Harrison H, Farnie G, Brennan KR, Clarke RB. Breast cancer stem cells: something out of notching? Cancer Res. 2010; 70:8973–6. https://doi.org/10.1158/0008-5472.CAN-10-1559. [PubMed]
13. Xie Y, Bayakhmetov S. PIM1 kinase as a promise of targeted therapy in prostate cancer stem cells. Mol Clin Oncol. 2016; 4:13–7. https://doi.org/10.3892/mco.2015.673. [PubMed]
14. Kolben T, Peröbner I, Fernsebner K, Lechner F, Geissler C, Ruiz-Heinrich L, Capovilla S, Jochum M, Neth P. Dissecting the impact of frizzled receptors in Wnt/β-catenin signaling of human mesenchymal stem cells. Biol Chem. 2012; 393:1433–47. https://doi.org/10.1515/hsz-2012-0186. [PubMed]
15. Zhang X, Lou Y, Wang H, Zheng X, Dong Q, Sun J, Han B. Wnt signaling regulates the stemness of lung cancer stem cells and its inhibitors exert anticancer effect on lung cancer SPC-A1 cells. Med Oncol. 2015; 32:95. https://doi.org/10.1007/s12032-014-0462-1. [PubMed]
16. Wang YH, Imai Y, Shiseki M, Tanaka J, Motoji T. Knockdown of the Wnt receptor Frizzled-1 (FZD1) reduces MDR1/P-glycoprotein expression in multidrug resistant leukemic cells and inhibits leukemic cell proliferation. Leuk Res. 2018; 67:99–108. https://doi.org/10.1016/j.leukres.2018.01.020. [PubMed]
17. Chen J, Li Y, Yu TS, McKay RM, Burns DK, Kernie SG, Parada LF. A restricted cell population propagates glioblastoma growth after chemotherapy. Nature. 2012; 488:522–6. https://doi.org/10.1038/nature11287. [PubMed]
18. Brooks MD, Wicha MS. Tumor twitter: cellular communication in the breast cancer stem cell niche. Cancer Discov. 2015; 5:469–71. https://doi.org/10.1158/2159-8290.CD-15-0327. [PubMed]

19. Matsui WH. Cancer stem cell signaling pathways. Medicine (Baltimore). 2016; 95:S8–S19. https://doi.org/10.1097/MD.0000000000004765. [PubMed]
20. Tadesse S, Yu M, Kumarasiri M, Le BT, Wang S. Targeting CDK6 in cancer: State of the art and new insights. Cell Cycle. 2015; 14:3220–30. https://doi.org/10.1080/15384101.2015.1084445. [PubMed]
21. Scheicher R, Hoelbl-Kovacic A, Bellutti F, Tigan AS, Prchal-Murphy M, Heller G, Schneckenleithner C, Salazar-Roa M, Zöchbauer-Müller S, Zuber J. CDK6 as a key regulator of hematopoietic and leukemic stem cell activation. Blood, The Journal of the American Society of Hematology. 2015; 125:90–101. https://doi.org/10.1182/blood-2014-06-584417. [PubMed]
22. Kishino E, Ogata R, Saitoh W, Koike Y, Ohta Y, Kanomata N, Kurebayashi J. Anti-cell growth and anti-cancer stem cell activity of the CDK4/6 inhibitor palbociclib in breast cancer cells. Breast Cancer. 2020; 27:415–425. https://doi.org/10.1007/s12282-019-01035-5. [PubMed]
23. Nishino K, Umezawa A. DNA methylation dynamics in human induced pluripotent stem cells. Hum Cell. 2016; 29:97–100. https://doi.org/10.1007/s13577-016-0139-5. [PubMed]
24. Czerwinska P, Kaminska B. Regulation of breast cancer stem cell features. Contemp Oncol (Pozn). 2015; 19:A7. https://doi.org/10.5114/wo.2014.47126. [PubMed]
25. Cazet AS, Hui MN, Elsworth BL, Wu SZ, Roden D, Chan CL, Skhinas JN, Collot R, Yang J, Harvey K. Targeting stromal remodeling and cancer stem cell plasticity overcomes chemoresistance in triple negative breast cancer. Nat Commun. 2018; 9. https://doi.org/10.1038/s41467-018-05220-6. [PubMed]
26. Salgado BS, Rocha RM, Soares FA, Gärtner F, Rocha NS. Snai-1 and Epithelial-Mesenchymal Transition-Related Protein Immunoexpression in Canine Mammary Carcinomas. Advances in Breast Cancer Research. 2014; 3:111–117. https://doi.org/10.4236/abcr.2014.34017.
27. Shankar J, Nabi IR. Actin cytoskeleton regulation of epithelial mesenchymal transition in metastatic cancer cells. PLoS ONE. 2015; 10:e0119954. https://doi.org/10.1371/journal.pone.0119954. [PubMed]
28. Schoenhals M, Kassambara A, De Vos J, Hose D, Moreaux J, Klein B. Embryonic stem cell markers expression in cancers. Biochem Biophys Res Commun. 2009; 383:157–62. https://doi.org/10.1016/j.bbrc.2009.02.156. [PubMed]
29. Charafe-Jauffret E, Ginestier C, Bertucci F, Cabaud O, Wicinski J, Finetti P, Josselin E, Adelaide J, Nguyen TT, Monville F. ALDH1-positive cancer stem cells predict engraftment of primary breast tumors and are governed by a common stem cell program. Cancer Res. 2013; 73:7290–300. https://doi.org/10.1158/0008-5472.CAN-12-4704. [PubMed]
30. Ginestier C, Hur MH, Charafe-Jauffret E, Monville F, Dutcher J, Brown M, Jacquemier J, Viens P, Kleer CG, Liu S. ALDH1 is a marker of normal and malignant human mammary stem cells and a predictor of poor clinical outcome. Cell stem cell. 2007; 1:555–67. https://doi.org/10.1016/j.stem.2007.08.014. [PubMed]
31. Lin F, Zhang H, Huang J, Xiong C. Substrate stiffness coupling TGF-β1 modulates migration and traction force of MDA-MB-231 human breast cancer cells *in vitro*. Biomater Sci Eng. 2018; 4:1337–45. https://doi.org/10.1021/acsbiomaterials.7b00835.
32. Le Magnen C, Bubendorf L, Ruiz C, Zlobec I, Bachmann A, Heberer M, Spagnoli GC, Wyler S, Mengus C. Klf4 transcription factor is expressed in the cytoplasm of prostate cancer cells. Eur J Cancer. 2013; 49:955–63. https://doi.org/10.1016/j.ejca.2012.09.023. [PubMed]
33. Oyinlade O, Wei S, Kammers K, Liu S, Wang S, Ma D, Huang ZY, Qian J, Zhu H, Wan J. Analysis of KLF4 regulated genes in cancer cells reveals a role of DNA methylation in promoter-enhancer interactions. Epigenetics. 2018; 13:751–68. https://doi.org/10.1080/15592294.2018.1504592. [PubMed]
34. Roy M, Pear WS, Aster JC. The multifaceted role of Notch in cancer. Curr Opin Genet Dev. 2007; 17:52–9. https://doi.org/10.1016/j.gde.2006.12.001. [PubMed]
35. Kotiyal S, Bhattacharya S. Breast cancer stem cells, EMT and therapeutic targets. Biochem Biophys Res Commun. 2014; 453:112–6. https://doi.org/10.1016/j.bbrc.2014.09.069. [PubMed]
36. Fu C, Lin R, Yu J, Chang W, Liao G, Chang W, Tseng L, Tsai Y, Yu J, Yu A. A novel oncogenic role of inositol phosphatase SHIP2 in ER-negative breast cancer stem cells: involvement of JNK/vimentin activation. Stem cells (Dayton, Ohio). 2014; 32:2048–60. https://doi.org/10.1002/stem.1735. [PubMed]
37. Liu S, Dontu G, Mantle ID, Patel S, Ahn NS, Jackson KW, Suri P, Wicha MS. Hedgehog signaling and Bmi-1 regulate self-renewal of normal and malignant human mammary stem cells. Cancer Res. 2006; 66:6063–71. https://doi.org/10.1158/0008-5472.CAN-06-0054. [PubMed]
38. Klahan S, Wu MS, Hsi E, Huang CC, Hou MF, Chang WC. Computational analysis of mRNA expression profiles identifies the ITG family and PIK3R3 as crucial genes for regulating triple negative breast cancer cell migration. Biomed Res Int. 2014; 2014:536591. https://doi.org/10.1155/2014/536591. [PubMed]
39. Barkan D, Chambers AF. β1-integrin: a potential therapeutic target in the battle against cancer recurrence. Clin Cancer Res. 2011; 17:7219–23. https://doi.org/10.1158/1078-0432.CCR-11-0642. [PubMed]
40. Guo L, Sun C, Xu S, Xu Y, Dong Q, Zhang L, Li W, Wang X, Ying G, Guo F. Knockdown of long non-coding RNA linc-ITGB1 inhibits cancer stemness and epithelial-mesenchymal transition by reducing the expression of Snail in non-small cell lung cancer. Thorac Cancer. 2019; 10:128–36. https://doi.org/10.1111/1759-7714.12911. [PubMed]
41. Chaffer CL, Marjanovic ND, Lee T, Bell G, Kleer CG, Reinhardt F, D'Alessio AC, Young RA, Weinberg RA. Poised chromatin at the ZEB1 promoter enables breast cancer cell plasticity and enhances tumorigenicity. Cell. 2013; 154:61–74. https://doi.org/10.1016/j.cell.2013.06.005. [PubMed]

42. Krebs AM, Mitschke J, Losada ML, Schmalhofer O, Boerries M, Busch H, Boettcher M, Mougiakakos D, Reichardt W, Bronsert P. The EMT-activator Zeb1 is a key factor for cell plasticity and promotes metastasis in pancreatic cancer. Nat Cell Biol. 2017; 19:518–29. https://doi.org/10.1038/ncb3513. [PubMed]
43. Chen HJ, Huang RL, Liew PL, Su PH, Chen LY, Weng YC, Chang CC, Wang YC, Chan MWY, Lai HC. GATA3 as a master regulator and therapeutic target in ovarian high-grade serous carcinoma stem cells. Int J Cancer. 2018; 143:3106–19. https://doi.org/10.1002/ijc.31750. [PubMed]
44. Shu J, Wu C, Wu Y, Li Z, Shao S, Zhao W, Tang X, Yang H, Shen L, Zuo X. Induction of pluripotency in mouse somatic cells with lineage specifiers. Cell. 2013; 153:963–75. https://doi.org/10.1016/j.cell.2013.05.001. [PubMed]
45. Bodenstine TM, Chandler GS, Seftor RE, Seftor EA, Hendrix MJ. Plasticity underlies tumor progression: role of Nodal signaling. Cancer Metastasis Rev. 2016; 35:21–39. https://doi.org/10.1007/s10555-016-9605-5. [PubMed]
46. Chambers I, Colby D, Robertson M, Nichols J, Lee S, Tweedie S, Smith A. Functional expression cloning of Nanog, a pluripotency sustaining factor in embryonic stem cells. Cell. 2003; 113:643–55. https://doi.org/10.1016/s0092-8674(03)00392-1. [PubMed]
47. Waghray A, Saiz N, Jayaprakash AD, Freire AG, Papatsenko D, Pereira CF, Lee DF, Brosh R, Chang B, Darr H. Tbx3 controls Dppa3 levels and exit from pluripotency toward mesoderm. Stem Cell Reports. 2015; 5:97–110. https://doi.org/10.1016/j.stemcr.2015.05.009. [PubMed]
48. Sang H, Wang D, Zhao S, Zhang J, Zhang Y, Xu J, Chen X, Nie Y, Zhang K, Zhang S. Dppa3 is critical for Lin28a-regulated ES cells naïve–primed state conversion. J Mol Cell Biol. 2019; 11:474–88. https://doi.org/10.1093/jmcb/mjy069. [PubMed]
49. Hoyt A, Moran A, Granger C, Sedani A, Saigh S, Brown J, Galoian K. PRP-1 significantly decreases the ALDHhigh cancer stem cell population and regulates the aberrant Wnt/β-catenin pathway in human chondrosarcoma JJ012 cells. Oncol Rep. 2019; 42:103–14. https://doi.org/10.3892/or.2019.7172. [PubMed]
50. Zhang S, Cui W. Sox2, a key factor in the regulation of pluripotency and neural differentiation. World J Stem Cells. 2014; 6:305–311. https://doi.org/10.4252/wjsc.v6.i3.305. [PubMed]
51. Zhu P, Wang Y, He L, Huang G, Du Y, Zhang G, Yan X, Xia P, Ye B, Wang S. ZIC2-dependent OCT4 activation drives self-renewal of human liver cancer stem cells. J Clin Invest. 2015; 125:3795–808. https://doi.org/10.1172/JCI81979. [PubMed]
52. Shi P, Liu W, Wang H, Li F, Zhang H, Wu Y, Kong Y, Zhou Z, Wang C, Chen W. Metformin suppresses triple-negative breast cancer stem cells by targeting KLF5 for degradation. Cell Discov. 2017; 3:170. https://doi.org/10.1038/celldisc.2017.10. [PubMed]
53. Kim CK, Saxena M, Maharjan K, Song JJ, Shroyer KR, Bialkowska AB, Shivdasani RA, Yang VW. Krüppel-like Factor 5 Regulates Stemness, Lineage Specification, and Regeneration of Intestinal Epithelial Stem Cells. Cell Mol Gastroenterol Hepatol. 2020; 9:587–609. https://doi.org/10.1016/j.jcmgh.2019.11.009. [PubMed]
54. Gilliland DG, Griffin JD. The roles of FLT3 in hematopoiesis and leukemia. Blood, The Journal of the American Society of Hematology. 2002; 100:1532–42. https://doi.org/10.1182/blood-2002-02-0492. [PubMed]
55. Kiyoi H. FLT3 Inhibitors. Chemotherapy for Leukemia. Springer. 2017; 167–79.
56. He X, Li S, Shi W, Lin Q, Ma J, Liu Y, Feng T, Cao X. Cyclin A1 is associated with poor prognosis in oesophageal squamous cell carcinoma. Oncol Lett. 2019; 18:706–12. https://doi.org/10.3892/ol.2019.10377. [PubMed]
57. Zhang G, Shang B, Yang P, Cao Z, Pan Y, Zhou Q. Induced pluripotent stem cell consensus genes: implication for the risk of tumorigenesis and cancers in induced pluripotent stem cell therapy. Stem Cells Dev. 2012; 21:955–64. https://doi.org/10.1089/scd.2011.0649. [PubMed]
58. Tang H, Chen B, Liu P, Xie X, He R, Zhang L, Huang X, Xiao X, Xie X. SOX8 acts as a prognostic factor and mediator to regulate the progression of triple-negative breast cancer. Carcinogenesis. 2019; 40:1278–87. https://doi.org/10.1093/carcin/bgz034. [PubMed]
59. Tudoran O, Soritau O, Balacescu L, Visan S, Barbos O, Cojocneanu-Petric R, Balacescu O, Berindan-Neagoe I. Regulation of stem cells-related signaling pathways in response to doxorubicin treatment in Hs578T triple-negative breast cancer cells. Mol Cell Biochem. 2015; 409:163–76. https://doi.org/10.1007/s11010-015-2522-z. [PubMed]
60. Loh YH, Wu Q, Chew JL, Vega VB, Zhang W, Chen X, Bourque G, George J, Leong B, Liu J. The Oct4 and Nanog transcription network regulates pluripotency in mouse embryonic stem cells. Nat Genet. 2006; 38:431–40. https://doi.org/10.1038/ng1760. [PubMed]
61. Kraus P, Sivakamasundari V, Yu HB, Xing X, Lim SL, Adler T, Pimentel JAA, Becker L, Bohla A, Garrett L. Pleiotropic functions for transcription factor zscan10. PLoS ONE. 2014; 9:e104568 https://doi.org/10.1371/journal.pone.0104568. [PubMed]
62. Avery S, Zafarana G, Gokhale PJ, Andrews PW. The role of SMAD4 in human embryonic stem cell self-renewal and stem cell fate. Stem Cells. 2010; 28:863–73. https://doi.org/10.1002/stem.409. [PubMed]
63. Deckers M, van Dinther M, Buijs J, Que I, Löwik C, van der Pluijm G, ten Dijke P. The tumor suppressor Smad4 is required for transforming growth factor β–induced epithelial to mesenchymal transition and bone metastasis of breast cancer cells. Cancer Res. 2006; 66:2202–9. https://doi.org/10.1158/0008-5472.CAN-05-3560. [PubMed]
64. Suzuki R, Fukui T, Kishimoto M, Miyamoto S, Takahashi Y, Takeo M, Mitsuyama T, Sakaguchi Y, Uchida K, Nishio A. Smad2/3 linker phosphorylation is a possible marker of cancer stem cells and correlates with carcinogenesis in a mouse model of colitis-associated colorectal cancer. J Crohns Colitis 2015; 9:565–74. https://doi.org/10.1093/ecco-jcc/jjv073. [PubMed]

65. Gong W, Sun B, Sun H, Zhao X, Zhang D, Liu T, Zhao N, Gu Q, Dong X, Liu F. Nodal signaling activates the Smad2/3 pathway to regulate stem cell-like properties in breast cancer cells. Am J Cancer Res. 2017; 7:503–517. [PubMed]

66. Kim ST, Sohn I, Do IG, Jang J, Kim SH, JUNG SH, Park JO, Park YS, Talasaz A, Lee J. Transcriptome analysis of CD133-positive stem cells and prognostic value of survivin in colorectal cancer. Cancer Genomics Proteomics. 2014; 11:259–66. [PubMed]

67. Tallack MR, Perkins AC. Three fingers on the switch: Krüppel-like factor 1 regulation of γ-globin to β-globin gene switching. Curr Opin Hematol. 2013; 20:193–200. https://doi.org/10.1097/MOH.0b013e32835f59ba. [PubMed]

68. Cotterman R, Knoepfler PS. N-Myc regulates expression of pluripotency genes in neuroblastoma including lif, klf2, klf4, and lin28b. PLoS ONE. 2009; 4:e5799. https://doi.org/10.1371/journal.pone.0005799. [PubMed]

69. Sun J, Lu Z, Deng Y, Wang W, He Q, Yan W, Wang A. Up-regulation of INSR/IGF1R by C-myc promotes TSCC tumorigenesis and metastasis through the NF-κB pathway. Biochim Biophys Acta Mol Basis Dis. 2018; 1864:1873–82. https://doi.org/10.1016/j.bbadis.2018.03.004. [PubMed]

70. Bal MG, Yukselten Y, Ozkanca S, Akgün E, Ugur HC, Sunguroglu A. Status of INSR, LRP1 and VCL transcripts on CD133+ Glioblastoma Multiforme Stem Cells. The FASEB Journal. 2016; 30:lb443–lb.

71. Pirozzi G, Tirino V, Camerlingo R, Franco R, La Rocca A, Liguori E, Martucci N, Paino F, Normanno N, Rocco G. Epithelial to mesenchymal transition by TGFβ-1 induction increases stemness characteristics in primary non small cell lung cancer cell line. PLoS ONE. 2011; 6:e21548. https://doi.org/10.1371/journal.pone.0021548. [PubMed]

72. Asiedu MK, Ingle JN, Behrens MD, Radisky DC, Knutson KL. TGFβ/TNFα-mediated epithelial–mesenchymal transition generates breast cancer stem cells with a claudin-low phenotype. Cancer Res. 2011; 71:4707–19. https://doi.org/10.1158/0008-5472.CAN-10-4554. [PubMed]

Supplementary Table 1: Annotation of stemness genes. See Supplementary Table 1

Supplementary Table 2: Sequence of primers and samples of investigated genes

Genes	Amplicon	Sequences
6p21.31 *OCT3*	144 bp	F 5′-ggattacagtcccaggacatc-3′<br>R 5′-gctgaataccttcccaaataga-3′<br>Probe FAM 5′-tgccaagctcctgaagcagaa-3′BHQ1
7q32.1 *SMO*	112 bp	F 5′-cgggactatgtgctatgtcag-3′<br>R 5′-ggttgatcttctccaccagaa-3′<br>Probe FAM 5′-accaagcagcccatccctgact-3′BHQ1
8q24 *MYC*	109 bp	F 5′-cagcagcgactctgaggag-3′<br>R 5′-agcagaaggtgatccagactct-3′<br>Probe FAM 5′-agaggcaggctcctggcaaa-3′BHQ1
q11.21 *SNAI2*	116 bp	F 5′-gaactggacacacatacagtgatta-3′<br>R 5′-cacacagtgatggggctgta-3′<br>Probe FAM 5′-ccatgcctgtcataccacaacca-3′BHQ1
9q31 *KLF4*	109 bp	F 5′-gggttttggcttcgtttct-3′<br>R 5′-caggtggctgcctcattaa-3′<br>Probe FAM 5′-cacctgaaccccaaagtcaacg-3′BHQ1
10p11.23 *BMI1*	94 bp	F 5′-tctttgaccagaacagattgg-3′<br>R 5′-gctgggcatcgtaagtatctt-3′<br>Probe FAM 5′-tcggaaagtaaacaaagacaaagaga-3′BHQ1
10p13 *VIM*	87 bp	F 5′-gcccttaaaggaaccaatga-3′<br>R 5′-gtcttggtagttagcagcttcaa-3′<br>Probe FAM 5′-tgcgtgaaatggaagagaactttg-3′BHQ1
13q12 *FLT3*	97 bp	F 5′-tcttgataacggatacagcatatc-3′<br>R 5′-ggtaaattgggcatcatcatt-3′<br>Probe FAM 5′-tcataagcaccagccaggagaatat-3′BHQ1
18q21.1 *SMAD2*	106 bp	F 5′-atgtgttaccataccaagcactt-3′<br>R 5′-ggtttgttcagagaagctgtaaa-3′<br>Probe FAM 5′-ccaaatacgatagatcagtgggataca-3′BHQ1
19p13.2 *KLF1*	166 bp	F 5′-tccaggtgtgatagccgag-3′<br>R 5′-gtatggcttctcccctgtgt-3′<br>probe fam 5′-tgcggcaagagctacaccaa-3′BHQ1
5p15.33 *TERT*	88 bp	F 5′-ttctggatttgcaggtgaac-3′<br>R 5′-catgcgtgaaacctgtacg-3′<br>Probe FAM 5′-tgcggcaagagctacaccaa-3′BHQ1
3q26.33 *SOX2*	126 bp	F 5′-acactgcccctctcacaca-3′<br>R 5′-gggttttctccatgctgttt-3′<br>Probe FAM 5′-tgggaggggtgcaaaagagg-3′BHQ1
Referee ACTB NM_001101.3	73 bp	F 5′- gagaagatgacccagatcatgtt -3′<br>R 5′- atagcacagcctggatagcaa -3′<br>Probe FAM 5′- agaccttcaacaccccagccat -3′BHQ1
Referee GAPDH NM_002046.3	124 bp	F 5′-gccagccgagccacatc-3′<br>R 5′-ggcaacaatatccactttaccaga-3′<br>Probe FAM 5′- cgcccaatacgaccaaatccg-3′BHQ1

Note: all samples — FAM → BHQ1; NM — RNA sequence number in the NCBI Nucleotide Database (http://www.ncbi.nlm.nih.gov/nuccore); bp — base pair; F — direct primer; R — reverse primer. All primers have original design.

Supplementary Figure 1: Heat map of copy number aberrations changes in a breast tumor during neoadjuvant chemotherapy. Note: red color - deletions; blue color - amplification. The top line is the patient (the notation corresponds to Table 1, for brevity, the letter part of the notation and the first digit are presented); white cell - before treatment, gray cell - after chemotherapy. The first column is the chromosome numbers and stemness genes; the second column is cytobands; cytobands for the localization of stemness genes are highlighted in green. See Supplementary Figure 1
