# Supplementary material for: Amplifications of stemness genes and the capacity of breast tumors for metastasis
Source: Oncotarget. 2020 May 26;11(21):1988–2001. doi: 10.18632/oncotarget.27608 (PMC7260118; doi:10.18632/oncotarget.27608)
Supplement: Supplementary file 2 [file oncotarget-11-1988-s002.pdf]

Supplementary Table 1: Annotation of stemness genes

Genes	Locus	Function
SOX2	3q26.33	The main regulator of the transcription factor, which controls pluripotency and self-renewal in embryonic stem cells, is involved in the induction of pluripotency, is part of the Yamanaki cocktail [1, 2].
DPPA2	3q13.13	The gene of pluripotency, which has specific expression in embryonic stem cells [3].
DPPA4	3q13.13	The product of this gene is nuclear factor that involved in stemm cell pluripotency and is necessary for embryogenesis. One of the most expression gene in embryonic stem cells [4].
GSK3B	3q13.33	*SNAIL1* and *NANOG* are one of the key stemness gene. The *SNAIL* activate *NANOG* through *SMAD1/AKT/GSK3B* pathway in lung cancer. [5].
TERT	5p15.33	*TERT* maintains the length and function of the telomere by adding telomeric repeats on the chromosome ends *de novo*. In normal tissues, it is expressed in stem cells and in progenitor cells. Almost always active in tumor cells [6].
BMP6	6p24.3	This gene encodes a secreted ligand of the TGF-beta superfamily of proteins. Recent studies have shown, *BMP6* has got an important role in self-renewal control and differentiation of stem cell, including mesenchymal stem cell (MSC), germinal stem cells (GSC), hematopoietic stem cells (HSC) and neural stem cells (NSCs) [7].
OCT4 (POU5F1)	6p21.33	Specifically expressed in mammalian totipotent embryonic stem cells and germ cells, playing a key role in the induction and maintenance of pluripotency. Included in Yamanaki cocktail [8].
SOX4	6p22.3	The ectopic overexpression of SOX4 in immortalized human mammary epithelial cells is sufficient to acquire mesenchymal features, increased invasion, and stem cell induction, as determined by the presence of a subpopulation of CD44high / CD24low cells. *SOX4* is induced by TGF-β in breast epithelial cells and was necessary for TGF-β-induced EMT [9].
NOTCH4	6p21.32	It was shown that enhanced expression of this oncoprotein in cultured mammary epithelial cells causes attachment-independent growth, invasion and loss of contact inhibition and induction of stem cells in breast cancer [10, 11]. Participates in the regulation of the hierarchy of stem and progenitor cells in normal and tumor tissues [12].
PIM1	6p21.2	*PIM1* plays an important role in the regulation of the cell cycle, cell proliferation, cell survival and multidrug resistance in cancer cells. PIM1 kinase plays the same role in the proliferation, self-renewal, and expansion of stem cells [13].
FZD9	7q11.23	It is expressed especially in muscles, kidneys, bones, testes, and the brain and plays an important role in maintaining stem cell populations in the blood, skin, and intestines. [14].
FZD1	7q21.13	The WNT Ligands Expression as well as *FZD1*, *FZD10*, *LRP5* and *LRP6*, was significantly increased in OCT4+ tumor cells [15]. It modulates the sensitivity to chemotherapeutic drugs in various types of human cancer and is associated with a poor prognosis [16].
WNT2	7q31.2	Circulating tumor cells released by tumors express the *WNT2* gene, which increases their stem properties and metastasis [17]. Tumors produce high levels of *WNT2, CXCL12* and *IL6*, which stimulates the self-renewal of tumor-initiating cells, which then produce more mesenchymal cells. *WNT2* is generated either by EMT-like CSCs or other more differentiated fixed EMT-like tumor cells [18]

SMO	7q32.1	Hyperexpression *SMO* increased frequency of stem cancer cells by 4 times and contributed further progression. Genetic knockdown of *SMO* in stem tumor cells resulted in complete elimination of tumor stem cells *in vivo* [19].
CDK6	7q21.2	Aberrant expression of *CDK6* protein has been observed in many cancer types [20, 21]. Pablocyclib (inhibitor *CDK6)* significantly reduces the proportion of tumor stem cells in various cell lines of breast cancer [22].
EPHA1	7q34-q35	The 8 genes were identified,that can be used as a new epigenetic markers in the assessment of iSC. In particular, the *EPHA1* gene is hypomethylated and highly expressed in iSC. [23].
SHH	7q36.3	The *SHH* signaling pathway promotes the self-renewal of stem and / or progenitor in various organs, and up-regulation of this pathway can contribute to oncogenesis and maintenance of the stem phenotype [24, 25].
SNAI2	8q11.21	The transcriptional repressor involved in the EMT of normal and tumor cells. For the latter, this occurs with invasion and intravation of blood vessels, in the process of hematogenous metastasis. *SNAI2* mRNA is expressed in the placenta, melanocytes, embryonic stem cells, leiomyosarcoma, neuroblastoma and glioblastoma. *SNAI2* a key regulator of nuclear transcription in breast tumor stem cells [26, 27].
MYC	8q24.21	It is a proto-oncogen encoding a protein that is a key transcriptional growth factor for normal and tumor stem cells. Included in Yamanaki cocktail [28].
ALDH1A1	9q21.13	It is one of the main markers of tumor stem cells of breast cancer and other localizations [29, 30].
TGFBR1	9q22.33	It is the prototype of a large family of secreted polypeptide growth factors, including bone morphogenetic proteins (BMPs), which regulate many cellular processes that affect proliferation, differentiation, migration, apoptosis, as well as maintaining the pluripotent stem state of cells [31].
KLF4	9q31.2	It plays a important role in cells growth, proliferation, differentiation, self-renewal of stem cell and maintaining pluripotency. Included in Yamanaki cocktail [32, 33].
NOTCH1	9q34.3	The *NOTCH* signaling pathway is an important form of intercellular communication, which plays a key role both in the management of the cell cycle, differentiation and formation of stem cell potential. The *NOTCH* gene family consists of transmembrane receptors - *NOTCH1*, *2, 3, 4* [34].
VIM	10p13	Vimentin expression is associated with epithelial-mesenchymal transition, invasion, migration, and metastasis. *VIM* expression has been shown to increase cell invasion and migration in breast cancer [37]. *VIM* increases the number of *ALDH1* + breast stem cells, their tumorigenicity and the formation of mammospheres [35, 36]
BMI1	10p12.2	He is a member of the Polycomb 1 (PcG) family. The Hedgehog pathway and *BMI1* are involved in embryonic development and self-renewal in stem cells and tumor cells of various locations, including breast tumors [25, 37].
ITGB1	10p11.22	*ITGB1* is involved in the regulation of the actin cytoskeleton, and is also closely associated with cell migration and growth. *ITGB1* is a key regulator of the transition from dormancy to active proliferation of tumor cells [38, 39]. It was shown that knockdown of the long non-coding RNA linc-*ITGB1*, activating the expression of *ITGB1*, inhibits the formation of

		OSCs and the expression of stem genes such as *SOX2, NANOG, OCT4, MYC* and CD133 [40].
ZEB1	10p11.22	*ZEB1* is a regulator of transcription, involved in the embryonic development and progression of cancer. *ZEB1* induces an epithelial-mesenchymal transition. Transfection of *ZEB1* leads to a stem transition of breast tumor cells. Link EMT with the formation of the stem phenotype [41, 42].
GATA3	10p14	*GATA3* can facilitate successful reprogramming into pluripotent stem cells and is essential for maintaining pluripotency. *GATA3* is highly expressed in ovarian cancer stem cells compared to progenitor cells and is associated with poor prognosis [43, 44].
NODAL	10q22.1	A member of the transforming growth factor beta (TGFβ) superfamily, *NODAL* is an established regulator of early embryonic development. *NODAL* is also an important factor in maintaining the pluripotency of embryonic stem cells through the regulation of major transcription programs [45].
NANOG	12p13.31	*NANOG* is a protein that regulates the functions of transcriptional regulation, self-renewal, and pluripotency. Together with *SOX2* and *OCT4, NANOG* plays a key role in maintaining the properties of embryonic stem cells [46].
DPPA3	12p13.31	It plays a role in cell division and maintaining cell pluripotency. Overexpression of *DPPA3* increases the proliferation rate of tumor cells and mammosphere formation, as well as migration and invasion [47]. *DPPA3* is a subordinate target for Lin28a in the induction of pluripotent state of cells [48].
CCND2	12p13.32	High expression of *CCND2* observed in ALDH high tumor stem cells [49]
SOX1	13q34	*SOX1* and *SOX2* are two closely related transcription factors belonging to a subgroup of the *SOXB1* family, which are largely involved in the regulation of pluripotent stem cells and nerve stem cells [50].
ZIC2	13q32.3	*ZIC2* was highly expressed in hepatic stem tumor cells, and in general, *ZIC2* plays a crucial role in the maintenance and survival of both normal stem cells and stem tumor cells [51].
KLF5	13q21.3	When *KLF5* expression is suppressed, the percentage of tumor stem cells of thrice-negative breast cancer is reduced [52]. *KLF5* provides epigenetic and transcriptional control of the *Wnt* and *NOTCH* pathway genes, and is crucial for maintaining stem stem intestinal stem cells [53].
FLT3	13q12.2	*FLT3* is a receptor of tyrosine kinase expressed by immature hematopoietic cells and is important for the normal development of stem cells in many organs, the immune system and hemoblastosis cancer stem cells [54, 55].
CCNA1	13q.13.3	Dysregulation of *CCNA1* is involved in carcinogenesis, progression and metastasis of many types of solid tumors [56]. The increasing of expression observed in tumor stem cells of many locations [57].
SOX8	16p13.3	The simultaneous activation of *SOX8* and *ZEB1* in breast cancer is associated with biological characteristics such as tumor size, lymph node infiltration, and TNM stage; moreover, it promotes the growth and migration of tumor cells and regulates the properties of tumor stem cells [58].
CCNF	16p13.3	The high expression was found in the study of signaling pathways associated with stem cells of triple-negative breast cancer Hs578T, in response to treatment with doxorubicin. OCT4 stimulates the expression

		of *NANOG* and cyclin F, both are protein phosphatase 1 (PP1) inhibitors, preventing Rb dephosphorylation and increasing cell proliferation [59, 60].
ZSCAN10	16p13.3	Essential for maintaining pluripotency of embryonic stem cells (ESCs) through interaction with *OCT4* and *SOX2*, which are markers of pluripotency for ESCs [61].
SMAD4	18q21.2	*SMAD4* translocates to the nucleus as a heterotrimeric complex *SMAD2 / SMAD3-SMAD4* after activation of the *TGFβ* family of receptors. Knockdown *SMAD4* effectively inhibits TGF-β-induced epithelial-mesenchymal transition of breast tumor cells. Human embryonic stem cells (ESCs) remain undifferentiated after *SMAD4* knockdown. *SMAD4* is required to stabilize the undifferentiated state of ESCs [62, 63].
SMAD2	18q21.1	*SMAD2*-positive cells are considered tumor stem cells in colon cancer [64]. *NODAL* signaling enhances the expression of *ALDH1, CD44, CD133, SOX2, OCT4* and *NANOG* by activating the *SMAD2/3* pathway, thereby increasing tumorigenicity and mammosphere formation ability of breast tumor cells [65].
SALL3	18q23	Recent studies have shown a relationship between the level of *SALL3* expression and carcinogenesis. *SALL3* expression is significantly increased in colon cancer stem cells CD133 +, compared with negative tumor cells CD133 [66]
KLF1	19p13.13	*KLF1* is able to activate the CD44 promoter, which is the main marker of stem cells. *KLF1* is a transcriptional activator. It is also a regulator of many components of the cell cycle mechanism [67].
KLF2	19p13.11	*MYC* is a positive regulator of *KLF2*, *KLF4*, *LIF* и *LIN28B* expression in human neuroblastoma. Maintaining the expression level of *KLF2, KLF4,* and *LIN28B* are independent mechanisms by which *MYC* promotes pluripotency, the genesis of neuroblastoma, and the biology of ESC and iPSC [68].
INSR	19p13.2	The ectopic overexpression of *INSR* promotes the growth, migration, invasion, oncogenesis and metastasis of squamous tumors. *MYC* binds directly to *INSR* promoters [69]. Increased expression of *INSR* is observed in glioblastoma CD133 + tumor stem cells compared with CD133 cells [70].
TGFB1	19q13.2	*TGFβ* promotes epithelial-mesenchymal transition (EMT), invasion and increased metastatic potential [72]. *TGFb* may lead to the induction of CSC from non-CSC [71]. The effect of *TGFβ* and *TNFα* on breast cancer cells induces cancer stem cells with increased self-renewal ability, tumorigenicity and increased resistance to oxaliplatin, etoposide and paclitaxel [72].

All annotated genes, in addition to participating in the stem phenotype induction and the role in stem cell self-renewal, are directly related to carcinogenesis and tumor progression. It is important to note that the up-regulation of all these genes leads to increased metastasis *in vivo* systems and increased tumorigenicity and mammosphere formation *in vitro* systems.
